# Surrogate Production of Eggs and Sperm by Intrapapillary Transplantation of Germ Cells in Cytoablated Adult Fish

**DOI:** 10.1371/journal.pone.0095294

**Published:** 2014-04-18

**Authors:** Sullip Kumar Majhi, Ricardo Shohei Hattori, Sheikh Mustafizur Rahman, Carlos Augusto Strüssmann

**Affiliations:** 1 Graduate School of Marine Science and Technology, Tokyo University of Marine Science and Technology, Minato, Tokyo, Japan; 2 Division of Molecular Biology & Biotechnology, National Bureau of Fish Genetic Resources, Dilkhusa, Lucknow, India; University of Nevada School of Medicine, United States of America

## Abstract

Germ cell transplantation (GCT) is a promising assisted reproductive technology for the conservation and propagation of endangered and valuable genetic resources. In teleost fish, GCT in adult gonads has been achieved only in male recipients, limiting greatly the usefulness of this technique in situations where both sexes need equal and timely attention for conservation and/or propagation. Here we describe a simplified GCT approach that ultimately leads to production of donor-derived eggs and sperm in considerably short time. Donor germ cells isolated from young pejerrey *Odontesthes bonariensis* (Atherinopsidae) were transplanted non-surgically through the genital papilla into the sexually mature gonads of Patagonian pejerrey *O. hatcheri* recipients whose gonads have been depleted of endogenous GCs by heat (26°C) and chemical treatment (four doses of Busulfan at 30 mg/kg and 40 mg/kg for females and males, respectively). Transplanted spermatogonial and oogonial cells were able to recolonize the recipients' gonads and produce functional donor origin eggs and sperm within 7 months from the GCT. We confirmed the presence of donor-derived gametes by PCR in 17% and 5% of the surrogate *O. hatcheri* fathers and mothers, respectively. The crosses between surrogate fathers and *O. bonariensis* mothers yielded 12.6–39.7% pure *O. bonariensis* and that between a surrogate mother and an *O. bonariensis* father yielded 52.2% pure *O. bonariensis* offspring. Our findings confirm that transplantation of germ cells into sexually competent adult fish by non-surgical methods allows the production of functional donor-derived eggs and sperm in a considerably short time. The methods described here could play a vital role in conservation and rapid propagation of endangered fish genetic resources.

## Introduction

Various assisted reproductive technologies have been devised to efficiently produce functional gametes and offspring from endangered species and commercially important animals that are difficult to breed in captivity [1]. These approaches include cryopreservation of gametes and embryos, induction of multiple ovulations, embryo transfer, *in vitro* gametogenesis, nuclear transfer, and germ cell transplantation (GCT), among others [2]. GCT provides also a unique system for studying the cellular and molecular events that regulate the sequential steps of gonadogenesis and gametogenesis [3]–[5]. There is particular interest in developing efficient methods of GCT for fish due to the growing concern with dwindling fisheries stocks and loss of species/genetic biodiversity due to over exploitation and environmental degradation [2]. The success of GCT largely depends on the availability of recipients that are completely or partially devoid of endogenous germ cells [4], [6]–[8]. The recipient gonads must be also genetically compatible with the donor species [9] but most recipients seem to present little or no rejection to the transplanted cells even if they are from relatively unrelated donors [10]–[14]. This fact makes possible to use domesticated strains and/or prolific species as recipients in GCT.

Several options for eradication of endogenous GCs in GCT recipients have been tested in mammals such as treatment with cytotoxic drugs like Busulfan [6], [9], [10], irradiation [15], cold ischemia [16] and hyperthermic treatment [17]. Two types of recipients have been experimentally tested for GCT in fish. Triploid animals have been used for production of donor-derived gametes in salmonids [18] taking advantage of the fact that they are generally, though not always, sterile [19], [20]; but see [21]. However, this strategy requires the long-term rearing of recipient animals until adult size as triploids can be produced only by manipulation of genetic events during or shortly after fertilization [19]. An alternative is the use of recipient fish which are depleted of endogenous GCs by chemical and heat-cytoablative treatments [22]–[25]. One advantage of this approach is that, when applied to adult, sexually competent animals, it obviates long-term rearing of hosts and allows surrogate generation of gametes within a relatively short time from GCT. For instance, in our previous study, recipients prepared with such strategy and transplanted with donor germ cells produced donor-derived functional gametes within 6 months, with germline transmission rates of 1.2–13.3% [13]. In that study, the recipients were prepared by rearing at a temperature of 25°C and by administration of two doses of Busulfan (40 mg/kg BW) at 4 weeks intervals [13], [22]. However, we observed that many females developed ulcerations shortly after Busulfan treatment and suffered increased mortality not observed in males.

The method of transplantation also has variants such as microinjection of GCs in the blastodisc of blastula stage embryos [5], into the coelomic cavity of hatchlings [8], [18], and directly into gonads of adults by surgical or non-surgical (intra-papillar) intervention [4], [13]. Regardless of their advantages and disadvantages, GCT by all methods and at all developmental stages has led to production of donor-derived functional gametes. However, there are obvious differences in the level of skills and equipment required to perform GCT by each of these methods, and some may be inapplicable in remote areas of the world where conservation efforts are probably more necessary. More importantly, they entail a fundamental difference in the time needed for production of surrogate gametes as previously mentioned, particularly for the comparison between GCT in embryos/hatchlings and in sexually competent adults.

GCT into the ovary of adult females has never been explored, thereby posing a constraint to the production of female gametes in situations where both sexes need equal attention for conservation and propagation. In this context, we re-evaluated the GCT procedure of Majhi et al. [13] and Lacerda et al. [4] for sexually competent adult fish in order to 1) optimize the thermo-chemical treatments for enhancing germ cell niche availability for GCT while minimizing the occurrence of pathologies in recipients and 2) to examine the suitability of thermo-chemically sterilized gonads to support the colonization, proliferation and differentiation of foreign GCs transplanted by non-surgical, intra-papillar intervention. More importantly, we performed GCT in female recipients in addition to males. Thus, using the same model species employed in our previous studies, the congeneric recipient Patagonian pejerrey *Odontesthes hatcheri* and donor species pejerrey *Odontesthes bonariensis,* here we report to the best of our knowledge the first demonstration of the functional viability of gametes from surrogate parents of both sexes produced by intra-papillar transplantation in adult fish. The simplified GCT approach described in this study ultimately leads to surrogate production of eggs and sperm in considerably short time, therefore being suitable for timely *in vivo* propagation of genetic resources.

## Materials and Methods

### Ethics statement

This study was approved by the Animal Ethics Committee of Tokyo University of Marine Science and Technology. All the fishes used in the experiments were handled according to the prescribed guidelines. During the study period, the fishes were sacrificed by anesthetic overdose and the gonads were excised.

### Experimental animals and rearing protocols

One year old Patagonian pejerrey *Odontesthes hatcheri* (mean body weight±SD of 37.35±16.8 g for males and 33.1±13.6 g for females) used as recipients in this study were procured from Yoshida Station, Field Science Center, Tokyo University of Marine Science and Technology. Fish were stocked in 200 L tanks at a density of 7.5 kg of fish per m^3^ and reared in flowing brackish water (0.2–0.5% NaCl) under a constant light cycle (15L9D) at the Aquatic Rearing Facilities of Tokyo University of Marine Science and Technology, Shinagawa Campus. The animals were acclimated for two weeks at 17°C prior to the thermo-chemical treatments. The GC donors were 4–5 months old specimens of the congeneric species pejerrey *O. bonariensis* (mean body weight±SD of 0.91±0.1 g for males and 0.8±0.03 g for females) produced in our laboratory. Donors were stocked in 200 L tanks at a density of 1.0 kg of fish per m^3^ and reared in flowing brackish water (0.2–0.5% NaCl) under a constant light cycle (15L9D) at temperature (25°C) until use. Both groups of animals were fed pelleted commercial diet four times per day to satiation.

### Recipient preparation

In this study, we reared animals at a slightly higher temperature (26°C) than in a previous study (25°C; see [22]) in order to promote faster gonadal GC degeneration. We also increased the number of injections from two to four to accelerate GC loss. On the other hand, we reduced the dosage of Busulfan (Sigma-Aldrich, St. Louis, MO, USA) for females (30 mg/kg body weight; B30) compared to males (40 mg/kg body weight; B40) because the highest dosage appeared to be toxic to females (see Introduction; ref. [22]). Each of the experimental groups had 80 fish of each sex. Females and males in the control group (B0; 30 fish of each sex) received only the vehicle DMSO at 1% Kg BW (Wako Pure Chemicals Ind., Osaka, Japan). The four injections were administered at the start (day 0) and at the 2^nd^, 4^th^, and 6^th^ weeks. For checking the permanency of the GC loss, animals were transferred from 26°C to 17°C at 8 weeks and reared for an additional period of 16 weeks (recovery period).

### Histological analysis of the gonads

For histological observations on the process of GC loss and the permanency of the GC deficiency, 10 males and 10 females were randomly sampled from each group at the 4^th^, 8^th^, and 24^th^ weeks (end of the recovery period). Animals were killed by an overdose of anesthesia and their body weight was recorded. The gonads were dissected, macroscopically examined, photographed using a digital camera, and weighed to the nearest 0.01g. The middle portion of the right and left gonads from each fish were then immersed in Bouin's fixative for 24 hours and preserved in 70% ethanol. Gonads were processed for light microscopical examination following routine histological procedures up to preparation of thin (5 µm) sections and staining with hematoxylin-eosin. About 100–150 serial histological sections from each fish were examined under a microscope at magnifications between 10-100X. The degree of histological degeneration and GC loss of each specimen was classified following the criteria of Majhi *et al*. [22] with minor modifications (Table 1).

**Table 1:** Histological criteria for classification of gonadal integrity/degeneration in adult Patagonian pejerrey *Odontesthes hatcheri* (modified from Majhi *et al*. 2009).

Males	Class	Females
Cysts of spermatogonia and other spermatogenic stages	**I**	Cysts of oogonia interspersed with oocytes at various stages of development
Only cysts of spermatogonia; efferent ducts may or not contain residual spermatozoa	**II**	Light hypertrophy of the ovigerous lamellae; oocytes are few and atretic
Cysts of spermatogonia are few and small	**III**	Few if any oocytes and greatly reduced number of oogonia
Absence of spermatogonia and any other germ cells	**IV**	Absence of oogonia and any other germ cells

### Vasa gene expression analysis

The degree of GC loss in *O. hatcheri* gonads was monitored by Real Time RT-PCR analysis of *vasa* mRNA expression according to the protocols of Majhi *et al*. [22]. Briefly, samples from the middle part of the gonads were collected from all groups at the same time of the histological samples and stored in RNA*later* (Sigma-Aldrich) at −80°C until further processing. RNA was extracted using Trizol (Invitrogen) according to manufacturer's protocol. cDNA was synthesized using oligodT primers and Superscript reverse transcriptase (Invitrogen). Primers for Real-Time RT-PCR (forward: 5′-CCTGGAAGCCAGGAAGTTTTC-3′; reverse 5′-GGTGCTGACCCCACCATAGA-3′) were designed using Primer Express (ver. 2.0; Applied Biosystems, Foster City, CA, USA). The Real Time PCRs were run in an ABI PRISM 7300 (Applied Biosystems) using *Power*SYBR Green PCR Master Mix in a total volume of 15 ul which included 25 ng of first strand cDNA and 5 pmol of each primer. *β*-actin (forward: 5′-CTCTGGTCGTACCACTGGTATCG-3′; reverse: 5′-GCAGAGCGTAGCCTTCATAGATG-3′) was analyzed as an endogenous control. Quantification was performed using the standard curve method with 4 points and the ABI Prism 7300 Sequence Detection Software (v.1.2; Applied Biosystems, Foster City, CA, USA).

### Isolation and labeling of donor germ cells for transplantation

Donor cells from male and female *O. bonariensis* were isolated based on the protocol described by Majhi *et al*. [13]. Briefly, fish were sacrificed by anesthetic overdose and the gonads were excised and rinsed in phosphate-buffered saline. The gonadal tissue was finely minced and incubated in a dissociating solution containing 0.5% Trypsin (Worthington Biochemical Corp., Lakewood, NJ), 5% Fetal Bovine Serum (JRH Biosciences, Lenexa, KS), and 1 mM Ca^2+^ in PBS for 2 hr at 22°C. The dispersed gonadal cells were sieved through a nylon screen (mesh size 50 µm) to eliminate non-dissociated cell clumps, suspended in a discontinuous Percoll (Sigma-Aldrich, St. Louis, MO, USA) gradient, and centrifuged at 200×*g* for 20 min at 20°C. The phase containing predominantly gonial cells (spermatogonia or oogonia) was harvested and the cells were rinsed and subjected to a cell viability test by the Trypan blue (0.4% w/v) exclusion assay. The PKH 26 Cell Linker kit (Sigma-Aldrich, St. Louis, MO, USA) was then used to label the cells to determine their localization inside the recipient gonads. The cells were labeled with the dye at the concentration of 8 µl per mL for 10 min at room temperature and the staining was stopped by addition of an equal volume of heat-inactivated fetal bovine serum. Labeled cells were rinsed three times to remove unincorporated dye, suspended in Dulbecco Modified Eagle Medium (Life Technologies, Rockville, MD) with 10% fetal bovine serum, and stored on ice until transplantation.

### Germ cell transplantation procedures

On termination of heat-Busulfan treatments at 8 weeks, the water temperature of the experimental tank was gradually decreased (1–2°C/day) to pretreatment condition. Once the rearing temperature reached 17°C, GCT was performed in 50 B40 males and 50 B30 females. Briefly, the fish were anesthetized in 200 ppm Phenoxyethanol (Wako Pure Chemicals Ind., Osaka, Japan) and held upside down onto an operation platform under a microscope where they received a constant flow of oxygenated, cool water containing 100 ppm of the anesthetic through the gills. To prevent desiccation, the surface of the fish was moisturized during the entire GCT procedure, which took about 8–10 min per fish on average. A micro syringe and fine glass needle were used to inject the cell suspension into the gonads through the genital papilla (Figure S2). Each individual was injected with 50 µl of cell suspension containing approximately 7.5×10^4^/µl, at a flow rate of approximately 10 µl/min. Trypan blue was added to the injection medium to allow visualization of the cell suspension inside the needle and leakage during/after transplantation. The genital opening of each fish was topically treated with 10% Isodine (Meijiseika Ltd., Tokyo, Japan) after the procedure and the fish were resuscitated in clean water.

### Fate of donor cells after transplantation

The fate of donor cells was assessed preliminarily by microscopical observation of the PKH 26-labeled cells in gonadal sections at 2, 4, 6, 8, and 24 weeks after injection. For this purpose, gonads were excised from 3–5 transplanted animals for each sampling, washed in PBS, fixed in 4% formaldehyde (Wako Pure Chemicals Ind., Osaka, Japan) overnight at 4°C, immersed in 15% sucrose (Sigma-Aldrich, St. Louis, MO, USA) for 2–3 hrs, embedded in O.C.T. compound, frozen using dry ice and stored at −80°C until actual sectioning. Cryostat (Leica CM 1500, Germany) sections with a thickness of 10 µm were made from representative portions of the gonads, air dried for 45–60 min at RT, coverslip mounted using 1–2 drops of scotch instant glue, and observed under a fluorescent microscope (Nikon Eclipse E600, Tokyo, Japan). Control sections were prepared using the gonads of animals not subjected to the transplantation procedure. Images were captured using a Pixera (Santa Clara, CA, USA) digital camera (Penguin 600CL) and software (Viewfinder/Studio).

The presence of donor-derived gametes in the GCT recipients was examined by molecular (PCR) analysis 7 months after transplantation. For the analysis of males, 10–30 µl of sperm was manually stripped by gentle abdominal pressure in areas around the genital papilla. In females, 30–50 eggs were collected from each female by cannulation. DNA from sperm and eggs was extracted by the standard phenol:chloroform protocol and subjected to PCR analysis with *O.bonariensis*-specific primers (forward: 5′-CAGTGCAGGTCCAGCATGGG-3′ and reverse: 5′-TGTTCCGCCTCAGTGCTTCAG-3′; amplicon size 386 bp) and *O. hatcheri*-specific primers (forward: 5′-ATGATCAGCAGCTGAGCCCACCTCC-3′ and reverse: 5′-TGTTCCGCCTCAGTGCTTCAG-3′; amplicon size 386 bp) that were designed based on the sequence of the first intron of the *amha* genes of these species using Genetyx Ver 8.2.1 (Genetyx Corp. Tokyo, Japan). Primers for β-actin indicated in *vasa* gene expression analysis were used as positive controls. The PCR reactions were run in a Mastercycler EP Gradient S (Eppendorf, Hamburg, Germany) and consisted of an initial denaturation at 94°C for 3 min, 30 cycles of 94°C for 30 sec, 70°C for 30 sec, and 72°C for 1 min, following elongation at 72°C for 5 min. PCR products were electrophoresed on an agarose gel (1%), stained with ethidium bromide, and photographed for later analysis.

### Artificial insemination and progeny analysis

Gametes taken from the surrogate parents at 7 and 11 months after the GCT were used in artificial insemination together with eggs and sperm from pure *O. bonariensis* mothers and fathers. The fertilized eggs obtained from each cross were incubated at 17°C for subsequent analysis of fertilization, hatching and germline transmission rates (%). The template DNA used in the germline transmission rate analysis was extracted from each progeny within 7–10 days after hatching and subjected to PCR analysis using both *O. bonariensis-*specific and *O. hatcheri*-specific sequences and conditions indicated above.

### Statistical analyses

Measured parameters were compared among the treatments by one-way analysis of variance (ANOVA) followed by the Tukey test whereas donor-derived germline transmission rates at 7 and 11 months after GCT were compared by the Fisher's exact test. Both statistical analyzes were performed with GraphPad Prism ver. 6.00 (GraphPad Software, San Diego, California, USA). Data are presented as mean ± SE and differences between groups were considered as statistically significant at *P*<0.05.

## Results

### Preparation of recipient fish

The survival rate of both sexes was high and the minimum rate (95%) was recorded for Busulfan-treated females (B30). There was a significant reduction in body weight between the 4^th^ and 8^th^ weeks for both treated females (B30) and males (B40), as the fishes apparently reduced food intake compared to the untreated controls. This was particularly noticeable after administration of the 3^rd^ dose on the 4^th^ week (Figure S1). However, the animals regained weight once the treatment was discontinued and they were returned to the normal temperature (17°C). External pathologies such as the ulcerations observed in previous studies were not detected in this study.

### Gonadal histology and fertility of males

**Figure 1:**
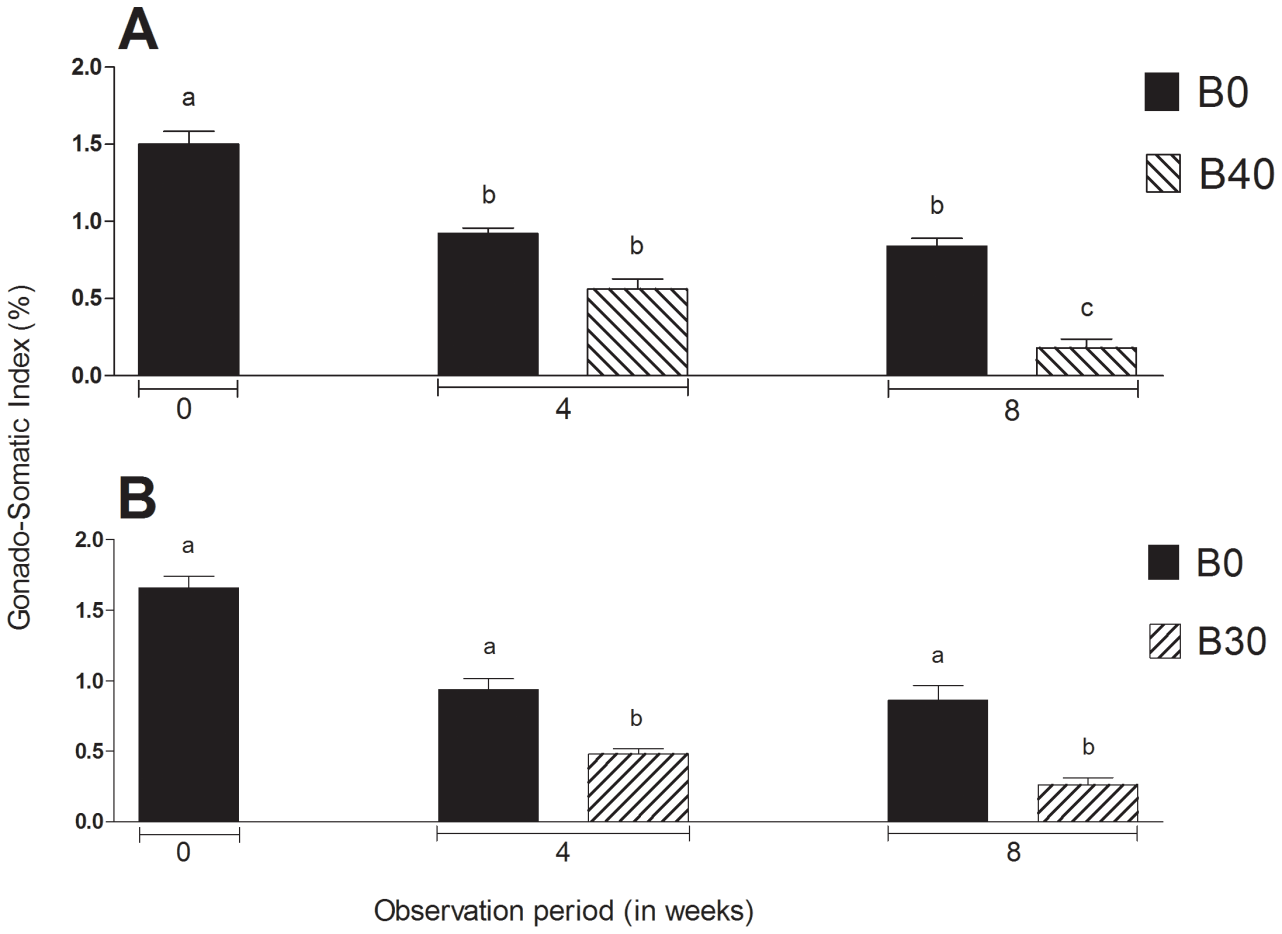
Changes in the gonado-somatic index of males (A) and females (B) subjected to heat (26°C) and Busulfan treatments (B0: Busulfan 0 mg/kg, controls; B30: 30 mg/kg, only females; B40: 40 mg/kg, only males) between 0 and 8 weeks. Columns with different letters vary significantly (ANOVA - Tukey test, *P*<0.05).

**Figure 2:**
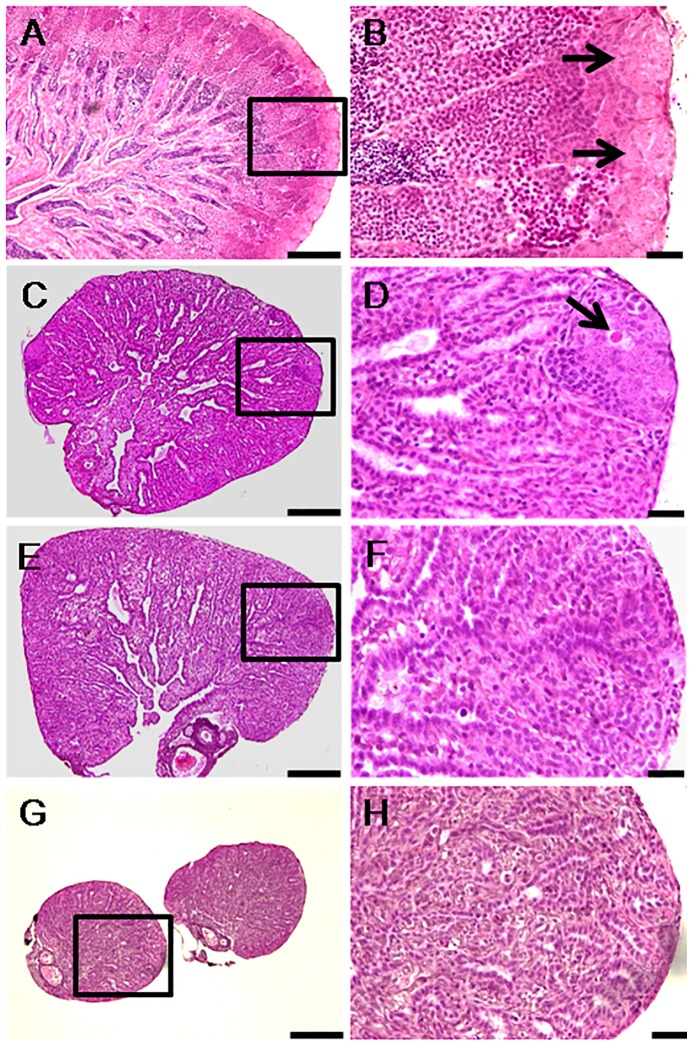
Histological changes in the testes of males subjected to heat (26°C) and Busulfan treatment (40 mg/kg). Panels on the right are high magnifications of insets in the left panels. A,B) Start of treatment (week 0; arrows indicate large cysts of spermatogonia in the blind end of the spermatogenic lobules; note also active spermatogenesis within the lobules). C,D) 4 weeks (arrow indicates an abnormal spermatogonium). E,F) 8 weeks (note the absence of spermatogonia). G,H) 24 weeks (note complete absence of GCs even after recovery for 16 weeks at 17°C). Scale bars indicate 100 µm (A, C, E, and G) and 20 µm (B, D, F and H).

**Figure 3:**
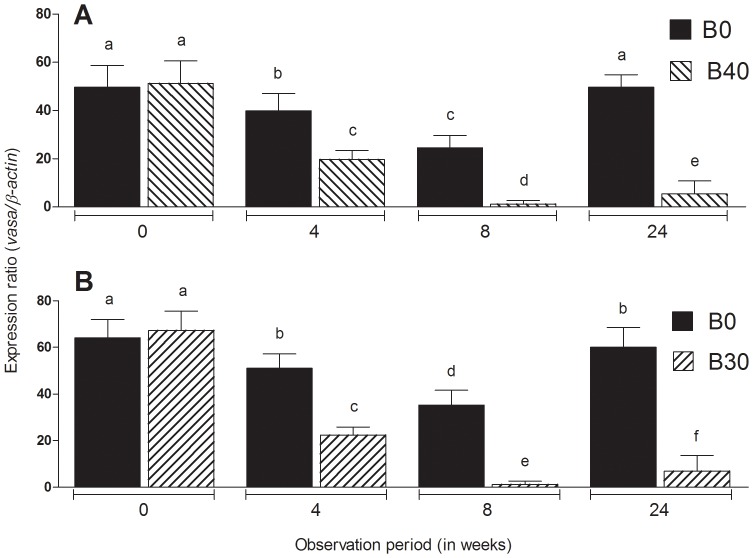
Changes in *vasa* gene transcript levels in the males (A) and females (B) subjected to heat (26°C) and Busulfan treatments (B0: Busulfan 0 mg/kg, controls; B30: 30 mg/kg, only females; B40: 40 mg/kg, only males) between 0 and 8 weeks and of Busulfan-treated animals after recovery for 16 weeks at 17°C (total 24 weeks). Columns with different letters vary significantly (ANOVA - Tukey test, *P*<0.05).

The gonado-somatic index (GSI) of males, including the controls (B0), decreased significantly between 0 and 4 weeks but only those in the group B40 continued decreasing further up to 8 weeks (Figure 1A). Microscopic examination of the gonads, on the other hand, revealed that all of the 10 control animals by 4 weeks and 5 out of 10 animals at 8 weeks had active spermatogenesis (Figure 2A–B; Table 1 and 2). The remaining 5 controls had relatively shrunk gonads and only cysts of spermatogonia. In contrast to the controls, B40 males at 4 weeks (total of 2 doses) had testes showing from absence of any spermatogenic stages beyond spermatogonia to the complete disappearance of all germ cells including spermatogonia. In addition, some testes presented conspicuous cysts of abnormal cells with variable size and staining properties ranging from dense basophilic (hematoxylin) to acidophilic (eosin) bodies (Figure 2C–D). At 8 weeks (total of 3 doses), the degree of germinal degeneration was far more severe and 9 out of 10 animals were found to be consistently devoid of GCs in all histological sections examined (Figure 2E–F). However, after 16 weeks of recovery at 17°C, presumed sterility was confirmed only in 4 out of 10 animals (Figure 2G–H; Table 2). The histological findings were corroborated by the Real Time RT-PCR analysis, which showed that *vasa* transcript levels were significantly lower in B40 males at 4 and 8 weeks compared to B0 controls (P<0.05; Figure 3A). Upon recovery for 16 weeks at 17°C, the transcript levels of B40 males remained low whereas those in B0 rebounded to pre-treatment levels.

**Table 2:** Frequency of individuals per category of histological appearance of the gonads in adult Patagonian pejerrey (*Odontesthes hatcheri*) subjected to heat (26°C) and Busulfan treatments (B0: Busulfan 0 mg/kg, controls; B30: 30 mg/kg, only females; B40: 40 mg/kg, only males) for germ cell depletion.

Treatments	Observation (weeks)	Sex	Histological category			
			I	II	III	IV
B0	4	♂	10	-	-	-
		♀	10	-	-	-
	8	♂	5	5	-	-
		♀	6	4	-	-
	24 (Recovery)	♂	10	-	-	-
		♀	10	-	-	-
B30	4	♀	-	5	5	-
	8	♀	-	-	2	8
	24 (Recovery)	♀	5	1	-	4
B40	4	♂	-	-	7	3
	8	♂	-	-	1	9
	24 (Recovery)	♂	5	-	1	4

Histological categories are described in Table 1.

### Gonadal histology and fertility of females

**Figure 4:**
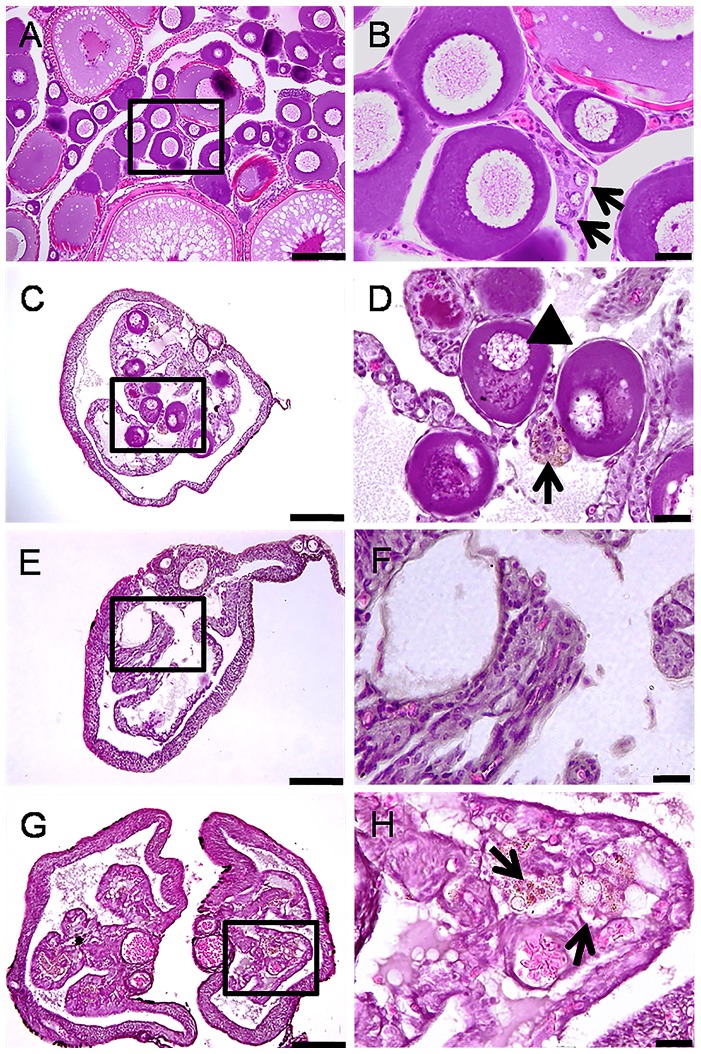
Histological changes in the ovaries of females subjected to heat (26°C) and Busulfan treatment (30 mg/kg). Panels on the right are high magnifications of insets in the left panels. A,B) Start of the experiment (week 0; arrows show a prominent cyst of oogonia; note also oocytes at various stages indicating active oogenesis). C,D) 4 weeks (note the absence of prominent cysts of oogonia, degenerating perinucleolar oocytes (arrowhead), and macrophage phagocytic activity indicated by deposition of yellowish-brown pigments (arrow). E,F) 8 weeks (note the absence of oogonia and other types of GCs). G,H) 24 weeks (note the absence of GCs, disorganized ovigerous lamellae (arrows), and hypertrophy of the tunica albuginea). Scale bars indicate 100 µm (A, C, E, and G) and 20 µm (B, D, F and H).

The GSI of B0 females did not vary significantly between 0 and 8 weeks whereas that of B30 showed significant decreases (Figure 1B). Control females did not show any marked histological changes throughout the study (Figure 4A-B) but 4 out of 10 individuals at 8 weeks showed light atrophy of the ovigerous lamellae, fewer oocytes, and presence of degenerating oogonia (Table 2). In the B30 group, a similar histological characteristic was observed in 5 individuals as early as 4 weeks. Furthermore, 5 out of 10 and 2 out of 10 females sampled at 4 and 8 weeks, respectively, had remarkable reduction in oogonial population as well as deposition of yellowish-brown pigments, an indication of the occurrence of macrophage phagocytic activity (Figure 4C–D) [24]. The remaining 8 females from group B30 that were examined at 8 weeks were completely devoid of oogonia in all sections examined (Figure 4E–F; Table 2). Following 16 weeks of recovery at 17°C, ovigerous lamellae were disorganized and oogonia were still missing altogether in 4 out of 10 females in this group (Figure 4G–H). The *vasa* transcript levels were significantly lower at 4 and 8 weeks in B30 females compared to the respective controls (P<0.05; Figure 3B). Upon recovery for 16 weeks, transcript levels recovered in the B0 but remain low in B30 group.

### Fate of the transplanted GCs in the recipient testes

**Figure 5:**
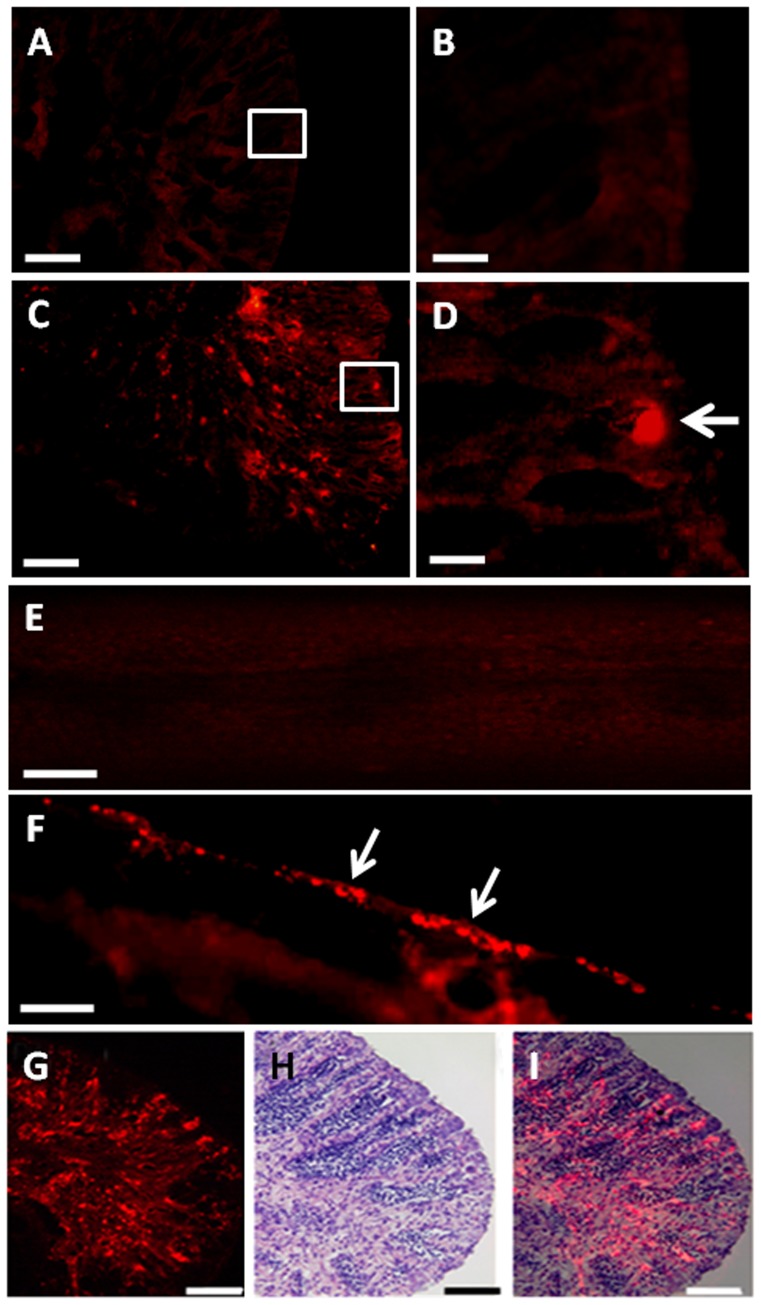
Fate of PKH-26-labeled donor germ cells in recipient testes between 4 and 24 weeks after germ cell transplantation. A,B) Cryostat section of a non-transplanted, control testis at 4 weeks showing the approximate location of the blind end of the spermatogenic lobules (B is a high magnification of the box in A). C,D) Cryostat section of a transplanted testis at 4 weeks showing the presence of transplanted GCs at the blind end of the spermatogenic lobules (arrow; D is a high magnification of the box in C). E) Whole-mount preparation of a non-transplanted control testis at 8 weeks. F) Whole-mount preparation of a transplanted testis at 8 weeks showing the presence of donor-derived GCs (arrows) along the length of the gonad. G-I) Cryostat (G), corresponding HE (H) and merged (I) sections of a transplanted testis at 6 months showing differentiation of the donor-derived cells along the spermatogenic lobules towards the efferent ducts. Scale bars indicate 100 µm (A, C, E, F, G, H and I) and 20 µm (B and D).

**Figure 6:**
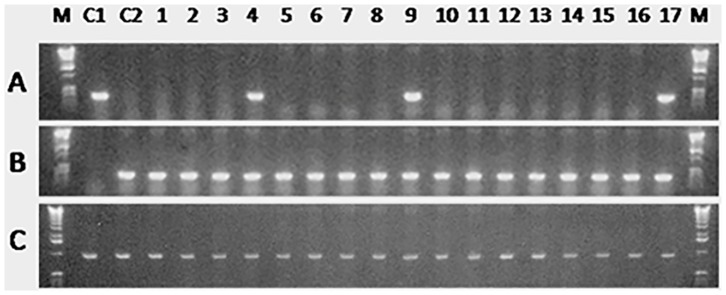
PCR analysis of sperm from 17 male recipients 7 months after germ cell transplantation. The primers used in the analysis were based on an *O. bonariensis*-specific sequence (A), an *O. hatcheri*-specific sequence (B), and β-actin (C) as a template control. Control lanes include pure *O. bonariensis* (C1) and *O. hatcheri* (C2) sperm. Donor-derived *O. bonariensis* spermatozoa were detected in the sperm of three surrogate *O. hatcheri* recipients shown in lanes 4, 9, and 17.

In males, the transplanted donor-derived GCs were found randomly distributed throughout the spermatogenic lobules in all 5 recipient males examined 2 weeks after transplantation (results not shown). At 4 and 6 weeks, a small number of donor cells (presumably spermatogonia stem cells) had reached the blind end of the lobules (cortical region of the testis; Figure 5C–D) in all animals examined. The transplanted GCs then proliferated and formed conspicuous cysts visible along the cortical region of the testis; this stage was observed in 4 out of 5 animals 8 weeks after transplantation (Figure 5F). After 6 months, donor germ cell cysts were undergoing differentiation and displacement towards the efferent ducts in 2 out of 5 recipients (Figure 5G–I). Sperm could be collected by gentle abdominal stripping in 20 out of 23 recipients examined 7 months after transplantation; the remaining 3 males were considered to be sterile based on histological analysis. Three of the spermiating animals were accidentally lost before sufficient sperm could be sampled for PCR analysis. PCR analysis of the remaining 17 GC-transplanted recipients revealed the presence of donor-derived cells together with endogenous cells in 17% (3 out of 17) (Figure 6). No recipient yielded pure donor-derived sperm in this study.

### Fate of the transplanted GCs in the recipient ovaries

**Figure 7:**
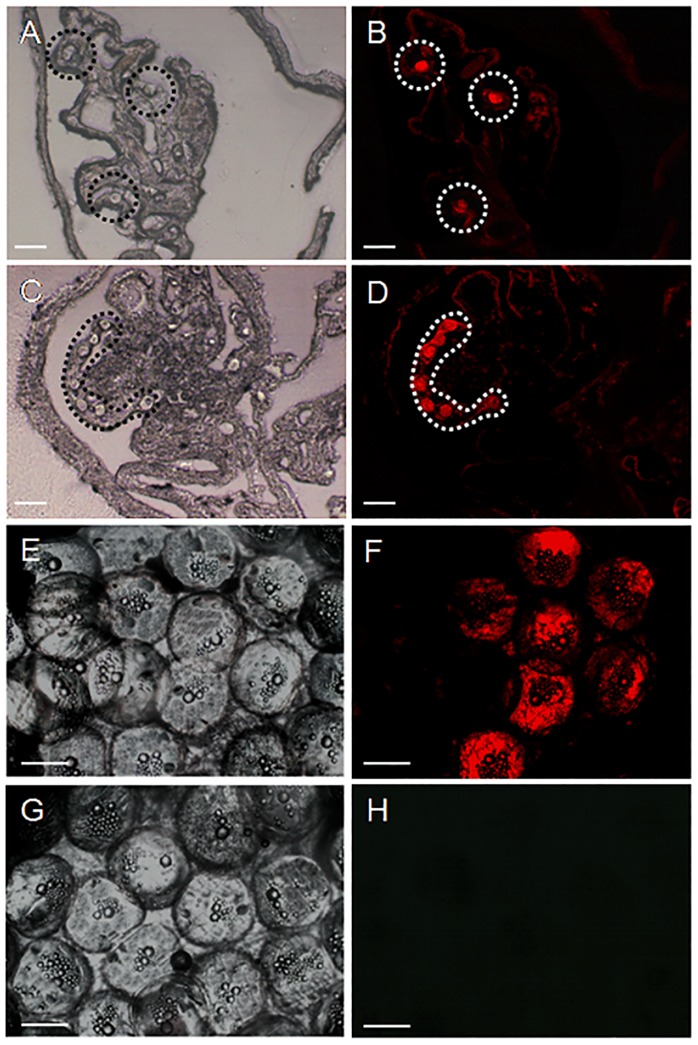
Fate of PKH 26-labeled donor germ cells in recipient ovaries between 2 and 24 weeks after germ cell transplantation. A,B) Cryostat section of a transplanted ovary at 2 weeks showing donor germ cells randomly distributed throughout the ovarian lamellae (circles; B is a fluorescent view of bright field A). C,D) Cryostat sections of a transplanted ovary at 4 weeks showing the donor germ cells (presumably oogonia) forming aggregations (highlighted; D is a fluorescent view of bright field C). E,F) Whole-mount preparation of oocytes from a transplanted female at 6 months showing the presence of fully differentiated donor-derived oocytes (characterized by retention of fluorescent label). G,H) Whole-mount preparation of oocytes from a non-transplanted control female. Scale bars indicate 20 µm (A, B, C and D) and 100 µm (E, F, G and H).

**Figure 8:**
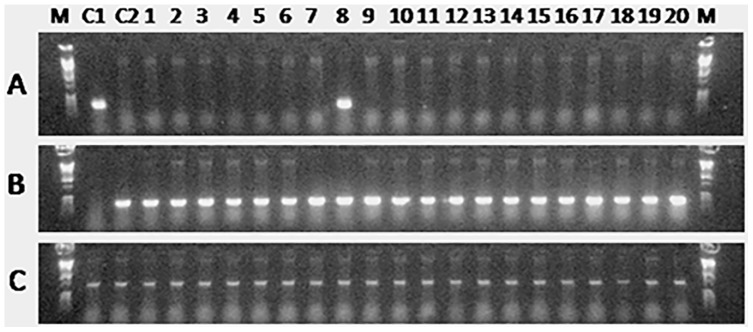
PCR analysis of eggs from 20 female recipients 7 months after germ cell transplantation. The primers used in the analysis were based on an *O. bonariensis*-specific sequence (A), an *O. hatcheri*-specific sequence (B), and β-actin (C) as a template control. Control lanes include pure *O. bonariensis* (C1) and an *O. hatcheri* (C2) eggs. Donor-derived *O. bonariensis* eggs were detected in one surrogate *O. hatcheri* recipient shown in lane 8.

The transplanted cells in females were randomly distributed throughout the ovarian lumen and the surface of the ovarian lamella during the first 2 weeks after GCT (Figure 7A–B). At 4 and 6 weeks, a small number of donor germ cells, presumably oogonia, formed clusters in the ovaries of 3 out of 5 recipients (Figure 7C–D). Larger clusters of oogonia were observed at 8 weeks and 6 months after GCT the transplanted cells had differentiated into mature oocytes in 1 out of 5 females examined (Figure 7E–F). At 7 months, ripe eggs were collected from recipients by intra-ovarian cannulation and the PCR analysis showed the presence of donor-derived cells along with endogenous cells in 5% (1 out of 20) ovulating recipients (Figure 8).

### Production of donor-derived offspring from surrogate parents

**Figure 9:**
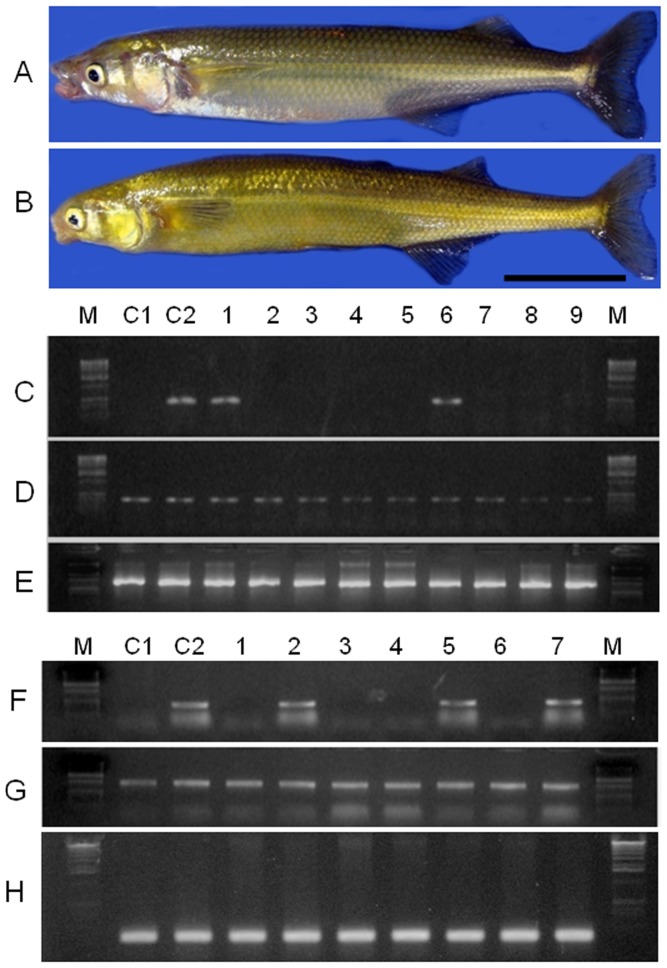
Model fish species used in this study (A: donor, *Odontesthes bonariensis*; B: recipient, *O. hatcheri*; scale bar indicates 1 cm) and results of PCR analysis of offspring from the crosses between surrogate *O. hatcheri* and pure *O. bonariensis* individuals. C –E) Results for the offspring of surrogate father (#2). The primers used in the analysis were based on an *O. hatcheri*-specific sequence (C), an *O. bonariensis*-specific sequence (D), and β-actin (E) as a template control. Control lanes include a pure *O. bonariensis* (C1) and a hybrid from an *O. hatcheri* father and an *O. bonariensis* mother (C2). Individuals shown in lanes 1 and 6 are hybrids and those shown in lanes 2–5 and 7–9 are pure, donor-derived *O. bonariensis*. F–H) Results for the offspring of surrogate mother (#1). Primers and control lanes follow the same order and codes as in panels C–E. Individuals shown in lanes 1, 3, 4, and 6 are pure donor-derived *O. bonariensis* and those shown in lanes 2, 5, and 7 are hybrids.

The GCT recipients found by PCR analysis to produce *O. bonariensis*-derived spermatozoa and eggs were then subjected to progeny testing by artificial insemination using eggs and sperm from pure *O. bonariensis* mothers and fathers 7 and 11 months after GCT. Such crosses produced viable offspring with normal fertilization and hatching rates compared to control animals. The crosses between surrogate fathers and *O. bonariensis* mothers yielded between 12.6 and 39.7% pure *O. bonariensis* in addition to hybrids between the two species; the donor-derived germline transmission rates 11 months after GCT did not show a clear trend of increase or decrease compared to those at 7 months (Table 3; Figure 9A–G). The cross between the surrogate mother and an *O. bonariensis* father yielded 52.2% and 39.7% pure *O. bonariensis* at 7 and 11 months, respectively, in addition to hybrids between the two species (Table 4).

**Table 3:** Results of artificial insemination of eggs derived from pure pejerrey mothers with sperm from three surrogate Patagonian pejerrey fathers (#1∼3; transplanted with pejerrey donor germ cells) and a control pure pejerrey father.

Father	Time after GCT (months)	Number of eggs (n)	Fertilization rate (n; %)	Hatching rate (n; %)	Donor-derived germline transmission rate (n; %)
#1	7	160	158(98.7)	150(94.9)	19(12.6)
	11	140	131(93.5)	122(93.1)	30(24.6)*
#2	7	155	147(94.8)	141(95.9)	56(39.7)
	11	170	162(95.2)	150(92.5)	44(29.3)
#3	7	130	125(96.1)	118(94.4)	38(32.2)
	11	155	146(94.1)	135(92.4)	28(20.7)*
Control	7	140	132(94.2)	125(94.6)	NA
	11	165	159(96.3)	148(93.0)	NA

Donor-derived germline transmission rates for each surrogate father were determined at 7 and 11 months after GCT; asterisks after the donor-derived germline transmission rate for 11 months indicate significant difference (Fisher's exact test) from the rate for the same father at 7 months. NA: not applicable

**Table 4:** Results of artificial insemination of eggs derived from a surrogate Patagonian pejerrey mother (#1; transplanted with pejerrey donor germ cells) and from a control pure pejerrey mother with sperm from a pure pejerrey father.

Mother	Time after GCT (months)	Number of eggs (n)	Fertilization rate (n; %)	Hatching rate (n; %)	Donor-derived germline transmission rate (n; %)
#1	7	108	98(90.7)	90(91.8)	47(52.2)
	11	120	108(90)	98(90.7)	39(39.7)
Control	7	105	100(95.2)	95(95.0)	NA
	11	130	123(94.6)	115(93.4)	NA

Donor-derived germline transmission rates determined for the same surrogate mother at 7 and 11 months after GCT were not significantly different (Fisher's exact test). NA: not applicable.

## Discussion

The efficiency of GC engraftment generally improves when the recipient gonads are devoid of endogenous GCs because of increased stem cell niche availability and accessibility to implanted cells [26], [27]. Multiple injections of Busulfan or repeated exposure to Gamma-ray radiation have been often used in adult mammalian and avian species to ablate the endogenous GCs prior to GCT [27]–[29]. In fish, injection of a single dose of Busulfan [30] or two dosages in association with elevated water temperature [22], [25] led to considerable depletion of GCs in sexually mature gonads, but complete sterility has never been achieved. Our previous study with sub-adult Patagonian pejerrey (*O. hatcheri*) showed that 2 doses of 40 mg Busulfan/kg BW and rearing at 25°C for 8 weeks led to marked depletion of GCs. However, the treatment did not result in complete sterility and caused development of ulcerations and increased mortality in females [22]. In this study we tested the efficiency of further (four) doses of Busulfan and increased temperature (26°C) to promote endogenous GC loss. We also tested a lower dosage for females (30 mg Busulfan/kg BW) compared to males (40 mg/kg BW) in an attempt to prevent the appearance of pathologies. The treatments resulted in high percentages of animals histologically classified as “sterile” at the end of the thermo-chemical treatment (8 weeks, 80–90% of the animals) and at the end of the recovery period at 17°C (6 months, 40% of the animals). These results compare well to none in our previous study [22], although the results of gamete collection at 7 months after GCT clearly indicate that only a few animals, about 13% of the males and no females, were completely sterile. Moreover, although we observed depressed food intake, leading to temporarily decreased body weight in both sexes, no other acute side effects and/or pathologies like ulcerations or deformities were observed. These observations confirm the suitability of multiple injection of Busulfan at concentrations of 30 mg/kg BW and 40 mg/kg BW for adult Patagonia pejerrey females and males, respectively, during recipient preparation for GCT. Further studies must test if the animals can tolerate a higher number of doses or temperature in order to increase the frequency of completely sterile individuals and, for that matter, if complete sterility is really best for GCT success compared to partial sterility (see [27]).

Since our report on the feasibility of surgical, intra-gonadal GCT in adult fish [13], GCT in sexually competent animals has been performed also in the tilapia model [4]. Lacerda's group was the first to produce donor fish sperm in surrogate fathers using intra-papillar GCT but the usefulness of this simple procedure has not been asserted in females. Here we confirm the usefulness of intra-papillar GCT in males of our fish model and report, to the best of our knowledge, the first evidence of donor GCs colonization and differentiation in the ovaries of female recipients as well as the functional viability of eggs produced by this method. Nevertheless, there was a significant difference between the sexes in the number of individuals which were successfully colonized by donor cells. For instance, 80% of male recipients had donor GCs occupying the basal compartment of spermatogenic lobules at 8 weeks whereas only 20% of the females had donor cells in the corresponding location (the ovigerous lamellae) at the same time. It remains to be determined if such difference in implantation rates between males and females reflects histological or physiological differences between the sexes. More likely, they may be the result of subtle differences in the degree of sterility or in the microenvironment conditions of the gonads [31] or even be an artifact due to the poor control over the number of GC stem cells injected into each individual [4]. In any case, the fact that even under suboptimal conditions the donor GCs have successfully colonized and undergone meiotic differentiation to produce gametes of donor origin in both sexes makes a powerful case for the simplicity and usefulness of intra-papillar GCT in adult fish.

Seven months after the procedure, donor-derived gametes were detected in 17% and 5% of the surrogate male and female, respectively, suggesting that the somatic cells of recipients' gonads have supported the proliferation and differentiation of donor GCs [9], [28], [32], [33]. Artificial insemination using the gametes from surrogate parents resulted in normal development of embryos with no noticeable abnormalities. We recorded donor-derived germline transmission rates of 12.6%-39.7% in three surrogate fathers and a surprising 52.2% in the surrogate mother 7 months after GCT. Of great interest is that the rates remained relatively stable between 7 and 11 months after GCT. These results compare favorably with germline transmission rates previously recorded for surgical GCT in adults of our fish model (1.2–13.3%; see [13]) and for transplantation of primordial germ cells (PGCs) into recipient embryos of salmonids (2–4%; see [34]) and trout (40–46%; see [31]). However, they are still well below the 100% donor-derived gametes produced by transplantation of PGCs into embryos neutered by either triploidy [18], [35], [36] or the use of an antisense dead end morpholino oligonucleotide [37]. As discussed above, it is tempting to conclude that the increased germline transmission rates in this study compared to our previous studies are the result of enhanced depletion of endogenous GCs prior to GCT. If this can be verified, it may be possible to enhance even further the germline transmission rates by additional optimization of the recipient preparation process and perhaps reach the 100% rates obtained for embryos.

In conclusion, the recipients produced in the present study proved to be able to host exogenous spermatogonia and oogonia cells that were implanted non-surgically through the genital papilla and support their differentiation into viable and functional gametes. Thus, the present approach using thermo-chemical treatments to prepare adult recipients for GCT in a short time could be a valuable alternative to methods that take considerably longer time and labor. The proposed combination of methods to prepare recipients and to perform GCT could be valuable in cases that require immediate attention such as species facing imminent extinction and for which suitable GCT hosts cannot be prepared in a timely fashion.

## Supporting Information

Figure S1 **Changes in mean body weight of males (A) and females (B) subjected to heat (26°C) and Busulfan treatments (B0: Busulfan 0 mg/kg, controls; B30: 30 mg/kg, only females; B40: 40 mg/kg, only males) between 0 and 8 weeks and of Busulfan-treated animals after recovery for 16 weeks at 17°C (total 24 weeks).** Columns with different letters vary significantly (ANOVA - Tukey test, *P*<0.05).
(TIF)

Figure S2 **Intra-papillar transplantation of donor cells into recipient gonads.** The recipients were placed onto an operation platform and received a constant flux of aerated anesthetic water through the gills during the procedure. The medium containing the donor cells was visualized by addition of Trypan blue during injection through the genital papilla (inset shows magnified view of injection). Scale bar indicates 1 cm.
(TIF)
