# Supplementary figures and images for: Surrogate Production of Eggs and Sperm by Intrapapillary Transplantation of Germ Cells in Cytoablated Adult Fish
Source: PLoS One. 2014 Apr 18;9(4):e95294. doi: 10.1371/journal.pone.0095294 (PMC3991631; doi:10.1371/journal.pone.0095294)

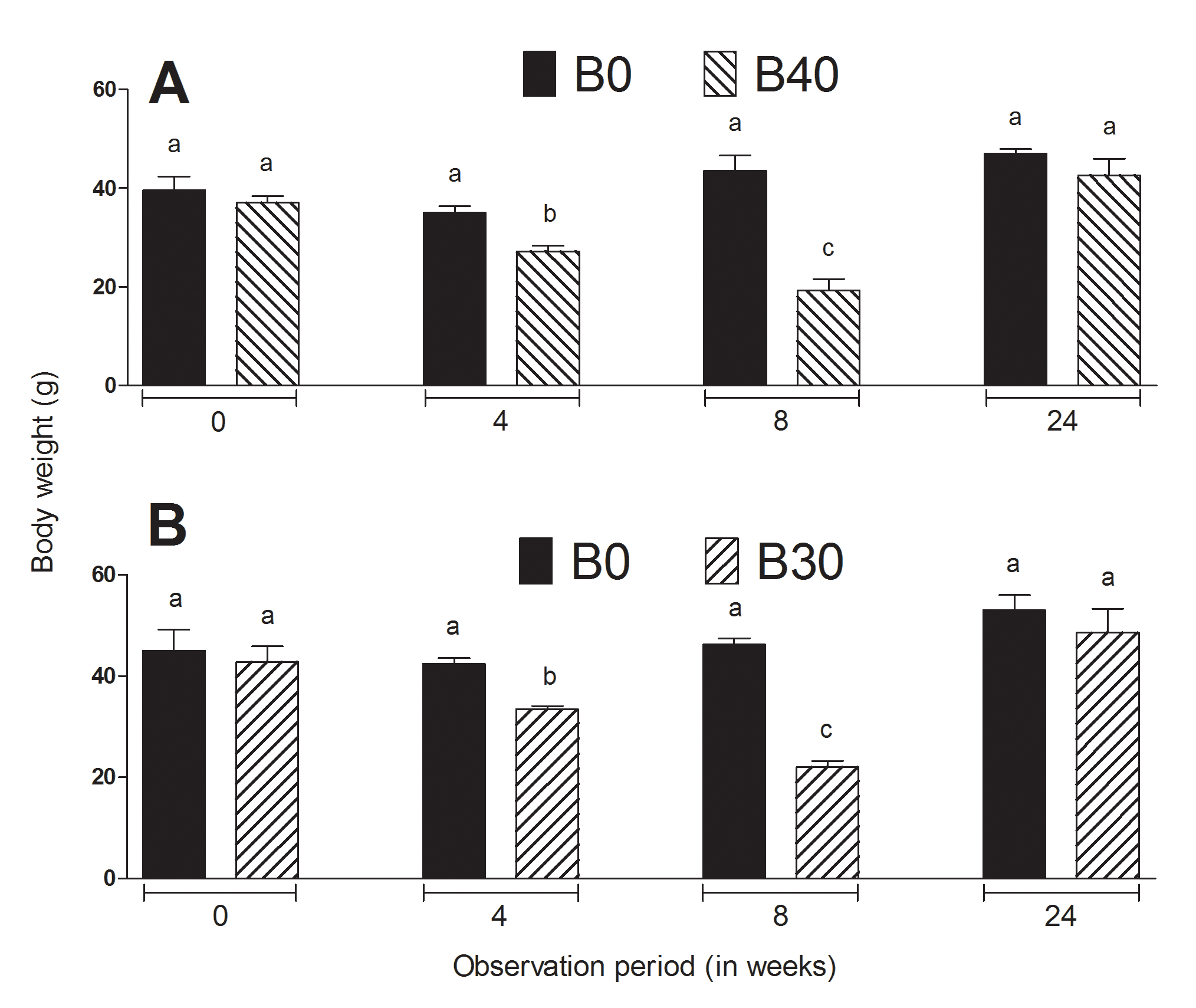

Supplement: Figure S1 — Changes in mean body weight of males (A) and females (B) subjected to heat (26°C) and Busulfan treatments (B0: Busulfan 0 mg/kg, controls; B30: 30 mg/kg, only females; B40: 40 mg/kg, only males) between 0 and 8 weeks and of Busulfan-treated animals after recovery for 16 weeks at 17°C (total 24 weeks). Columns with different letters vary significantly (ANOVA - Tukey test, P<0.05). (TIF) [file pone.0095294.s001.tif]

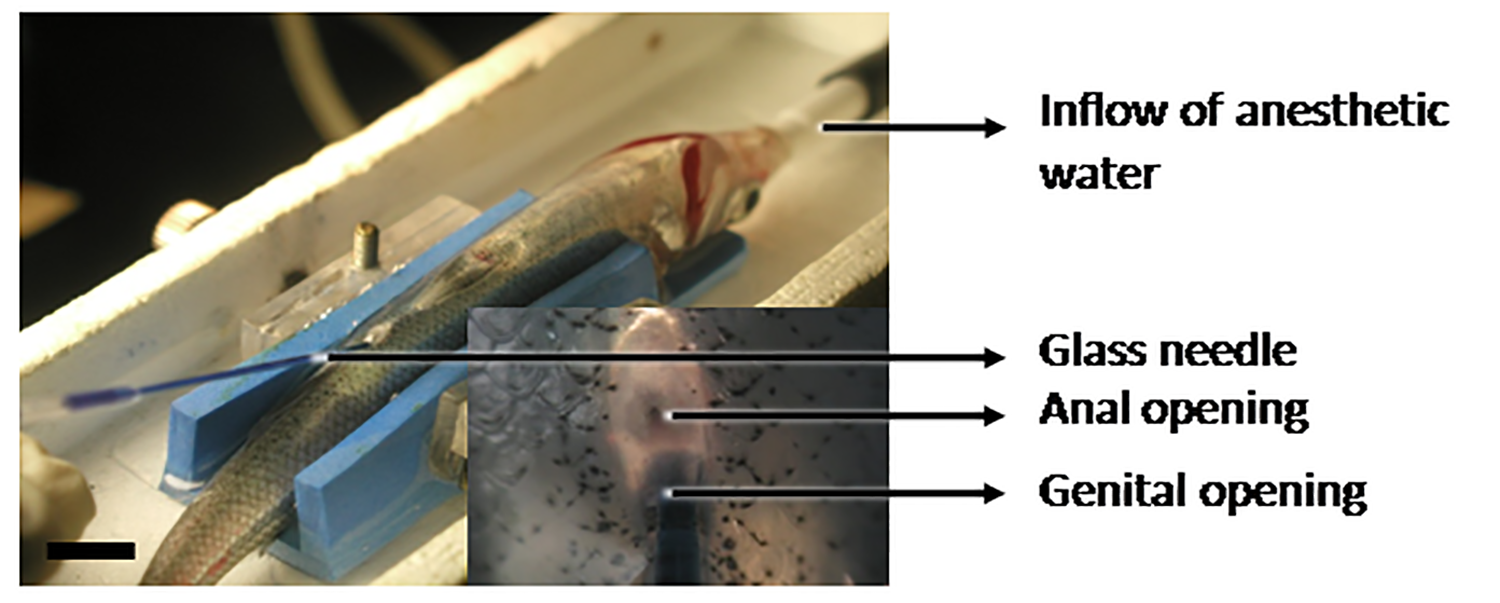

Supplement: Figure S2 — Intra-papillar transplantation of donor cells into recipient gonads. The recipients were placed onto an operation platform and received a constant flux of aerated anesthetic water through the gills during the procedure. The medium containing the donor cells was visualized by addition of Trypan blue during injection through the genital papilla (inset shows magnified view of injection). Scale bar indicates 1 cm. (TIF) [file pone.0095294.s002.tif]
